# Sentinel Lymph Node Biopsy in Prostate Cancer Patients: Results From an Injection Technique Targeting the Index Lesion in the Prostate Gland

**DOI:** 10.3389/fmed.2022.931867

**Published:** 2022-09-02

**Authors:** Lluís Fumadó, Jose M. Abascal, Antoni Mestre-Fusco, Sergi Vidal-Sicart, Guadalupe Aguilar, Nuria Juanpere, Lluís Cecchini

**Affiliations:** ^1^Department of Urology, Hospital del Mar, Barcelona, Spain; ^2^Department of Nuclear Medicine, Hospital Universitari de Girona Dr. Josep Trueta, Girona, Spain; ^3^Department of Nuclear Medicine, Hospital Clínic i Provincial, Barcelona, Spain; ^4^Department of Radiology, Hospital del Mar, Barcelona, Spain; ^5^Department of Pathology, Hospital del Mar, Barcelona, Spain

**Keywords:** prostate cancer, sentinel lymph node, radio-guided surgery, pelvic lymph node dissection, tracer deposition

## Abstract

**Objectives:**

To determine the accuracy of nodal staging in patients with prostate cancer (PCa) when ^99^ *^m^*Tc-nanocolloid radiotracer is injected into an index lesion (IL).

**Methods:**

This prospective study was conducted at our institution between June 2016 and October 2020. It included 64 patients with localized PCa with at least a 5% possibility for lymph node involvement in the Memorial Sloan Kettering Cancer Center nomogram, suitable for surgical treatment. All patients underwent magnetic resonance imaging (MRI) with IL and were pathologically confirmed. The day before surgery, transrectal ultrasound-guided injection (TRUS) of ^99^ *^m^*Tc-nanocolloid into the IL was performed. Surgical procedures included radical prostatectomy (RP), sentinel lymph node biopsy (SLNB), and extended pelvic lymphadenectomy (ePLND). Analysis was performed, including histopathological findings of RP, ePLND, and SLNB. The sensitivity, specificity, positive predictive value (PPV), negative predictive value (NPV), false negative (FN), false positive (FP), diagnostic yield, and non-diagnostic rate were calculated.

**Results:**

A total of 1,316 lymph nodes were excised, including 1,102 from the ePLND (83.7%) and 214 (16.3%) sentinel lymph nodes (SLN). 26 SLN were dissected outside the ePLND template. The final pathology demonstrated 46 (3.5%) lymph node metastasis, 31 (67.4%) in the SLNB and 15 (32.6%) in the non-SLN ePLND. At the patient level, 18 (28.1%) patients had pN1. With a mean follow-up of 33.1 months, 4/19 (21.1%) pN1 patients had undetectable PSA, and 3/19 (15.8%) had a PSA < 0.1 ng/mL. Lymph node dissection included 20.6 lymph nodes per patient (IQR 15–24.2), with 3.3 SLNB nodes per patient (IQR 2–4.2). PPV and NPV were 100 and 97.8%, respectively. Sensitivity and specificity were 94.4 and 100%, respectively. FN was 5.5% and FP was 4.3%. Diagnostic yields were 95.3% and the non-diagnostic rate was 4.7%.

**Conclusion:**

Radiotracer injection into the prostate IL offers promising results for staging purposes in cases in which ePLND is considered. Negative SLNB is a predictor of negative ePLND. Patients with a limited burden of nodal metastasis have a significant chance of remaining free of biochemical recurrence at mid-term follow-up.

## Introduction

Extended pelvic lymphadenectomy (ePLND) is the most reliable and accurate method for lymph node staging in prostate cancer (PCa) (1, 2); however, oncological benefits are controversial, and the surgical procedure is associated with potential complications (3, 4).

In 2003, Wawroschek et al. introduced the concept of sentinel lymph node biopsy (SLNB) for PCa to avoid the invasiveness and morbidity of large dissection templates (5). After a wide experience in this setting, a systematic analysis revealed that SLNB has comparable diagnostic accuracy for identifying lymph node metastasis to ePLND, while removing fewer lymph nodes (3) and current evidence shows the potential of SLNB for improving biochemical recurrence and clinical recurrence without increasing complications (6, 7). However, SLNB for PCa is still considered experimental (8).

Studies of lymphatic drainage of the prostate gland have revealed that it is highly variable and complex (9). Greater clarity regarding the lymphatic landing sites may be achieved by intraprostatic injection of ^99^ *^m^*Tc-nanocolloid combined with the intraoperative use of a gamma probe (9). Although PCa is a multifocal disease, there is increasing evidence that the largest and highest-grade tumor focus within the prostate, the index lesion (IL), drives the natural history of PCa (10). Our goal was to analyze the effectiveness of a modified injection of ^99^ *^m^*Tc-nanocolloid to the IL in the prostate in patients suitable for radical prostatectomy (RP), ePLND, and SLNB. This can improve the SLNB technique.

## Patients and Methods

### Patients

From June 2016 to October 2020, we conducted a prospective study on a cohort of 75 consecutive patients who underwent laparoscopic radical prostatectomy (LRP) and ePLND at our center. Inclusion criteria were as follows: localized intermediate- to high-risk PCa with at least 5% probability of lymph node involvement using the Memorial Sloan Kettering Cancer Center PCa nomogram (11); a multiparametric prostate magnetic resonance imaging (MRI) with at least one suspected tumor lesion according to Prostate Imaging Reporting and Data System (PI-RADS) V2.0 criteria of grades 3, 4, and 5; and histopathologic confirmation of the IL (higher grade and/or volume). Exclusion criteria were as follows: patients without preoperative MRI, MRI with no defined IL, targeted prostate biopsy not confirmative of prostate adenocarcinoma, and/or patients who did not consent to participate.

The staging workup consisted of a bone scan, chest and abdominal computed tomography (CT) scan, and pelvic MRI in all cases. Prostate biopsies were all performed through the cognitive fusion technique, transrectally or transperineally (at least 12-core systematic random biopsies and then 2–4 specific targeted biopsies to the IL). Three cases were excluded because no preoperative MRI was available, five due to the absence of any identifiable PI-RADS lesion, and three who did not consent to participate. A total of 64 patients were included in the analysis.

All procedures were performed in accordance with the ethical standards of the institutional and/or national research committee and with the Helsinki Declaration and its later amendments or comparable ethical standards. All patients provided written informed consent, and the study was approved by the institutional ethical committee (CEIm-Parc de Salut MAR code 8607).

### Radiotracer Injection and Surgical Technique

The day before surgery, in the afternoon, a radiotracer dose was prepared according to the protocol. ^99^ *^m^*Tc-nanocolloid was prepared by adding sodium pertechnetate (approximately 1,000 MBq, range 740–1,500 MBq) in 1 mL saline to a commercial vial of 0.5 mg human serum albumin nanocolloid (Nanocoll→; GE Healthcare, Eindhoven, the Netherlands). This preparation resulted in a nanocolloid concentration of 27:1 (range 20:1–40:1). After 30 min of incubation at room temperature, the radiotracer dose (225–296 MBq of ^99^ *^m^*Tc-nanocolloid in 0.6-cc volume, range 0.5–0.7 cc) was prepared and injected only to the IL and its peripheral area through a cognitive-fusion technique guided by transrectal ultrasound (TRUS). The total dose was split into 2–4 injections. All procedures were performed under current good manufacturing practices and under the supervision of the institution’s pharmacist.

**FIGURE 1:**
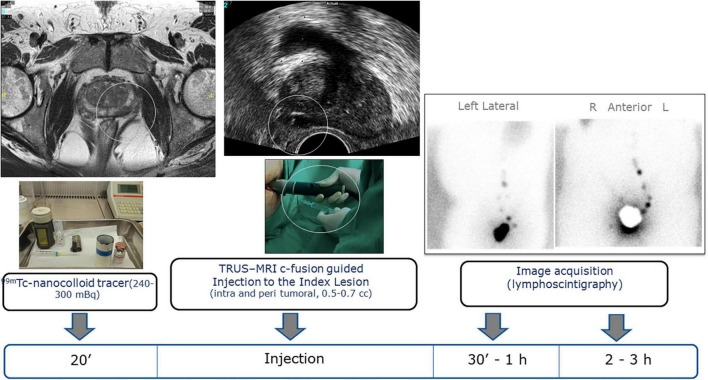
Transrectal ultrasound injections of diluted ^99^ *^m^*Tc-nancolloid to the index lesion and peripheral area before surgery. Lymphoscintigraphy, 1st day: Transrectal guided injection (TRUS) injection of diluted ^99^ *^m^*Tc-nanocolloid to the index lesion and peripheral area. MRI T2 weighted images showing a left lobe lesion (PZ). TRUS intralesional in the left lobe lesion. Lymphoscintigraphy images showing a left drainage predominantly in obturator fossa and external iliac artery area.

Lymphoscintigraphy was subsequently performed using a dual-head gamma camera (Symbia E-Cam, Siemens, Erlangen, Germany). Planar images were taken 30 min and 3–4 h post-injection. A methacrylate flood source filled with 74 MBq of ^99^ *^m^*Tc-nanocolloid was used for the body contour depiction. Sentinel lymph node dissection (SLND) mapping was based on preoperative lymphoscintigraphy imaging, identifying sentinel lymph nodes (SLN) as hot spots (Figure 1).

Surgical procedures included LRP with SLNB and ePLND. SLNB was the first procedure, followed by ePLND, and finally LRP. In cases where the SLNB was very close to the prostate, the procedure began with the LRP to potentially avoid the shine-through effect and facilitate SLN identification.

**FIGURE 2:**
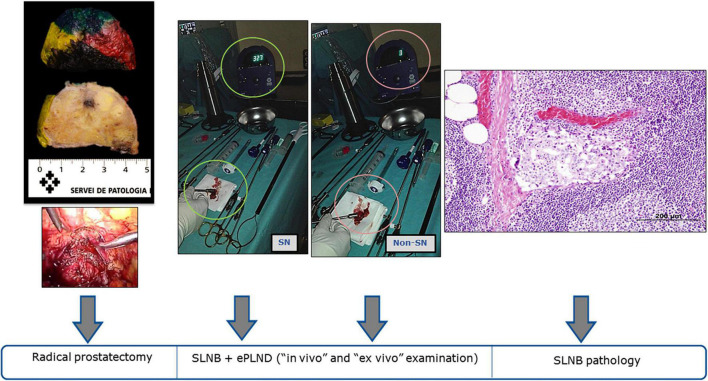
Surgery, 2nd day: Laparoscopic radical prostatectomy, sentinel node detection, extended pelvic lymphadenectomy and *ex vivo* confirmation. Surgery, 2nd day: Laparoscopic radical prostatectomy, Sentinel Node detection (SLNB), extended pelvic lymphadenectomy (ePLND) and pathology results of the tissue removed. Prostatectomy specimen, formalin-fixed and perpendicularly sectioned from apex to base. The index tumor lesion is located in the left lobe. Microscopic image of a SLN section showing a subcapsular metastasis of acinar prostatic adenocarcinoma (Hematoxylin-Eosin, 100x).

A laparoscopic hand-held gamma probe (Navigator; USSC, Norwalk, CT, United States) compatible with a 10-mm diameter laparoscopic trocar was used for acoustic gamma tracing. After anatomical exposition of the pelvic area, a gamma probe was introduced through the port, and SLN detection was conducted. Lymphoscintigraphic images were used to guide the gamma-probe scanning process (Figure 2).

During surgery, a lymph node was considered to be an SLN when: (a) it was the first node to be visualized on lymphoscintigraphy or showed increased activity on later images and (b) it was the most active node during surgery as determined by gamma tracing.

ePLND was performed according to the template described by Heidenreich et al. (medial border by the bladder and internal iliac artery, lateral border by the lateral aspect of the external iliac artery, distal border by the inguinal ligament, and proximal border by the ureteral crossing of the common iliac artery, including all the tissues in the obturator fossa) (12). At the end of the ePLND procedure, the gamma probe scans the surgical bed to detect any residual activity and potential SLN. The activity of all excised SLNs was assessed “ex vivo.” The number and location of the SLN were recorded.

### Histopathological Analysis

The retrieved SLNs and ePLND were sent to the pathology department, where lymph nodes were measured and sectioned perpendicular to their main axis in 2–3-mm-thick slices. All slices were fixed in 4% buffered formaldehyde for 12–72 h and completely embedded in paraffin. Hematoxylin and eosin-stained 3–4 μm sections of the lymph nodes were examined by an expert pathologist (NJ). In two cases, step sections were performed to confirm the absence of metastases.

### Measured Outcomes and Statistical Analysis

Patient characteristics (categorical data) were presented as absolute values and percentage frequencies. The number of SLN removed and their intraoperative locations were also recorded. Analysis was performed, including tumor characteristics, SLN drainage, and histopathologic findings of ePLND as well as SLN. *Sensitivity* (probability of being diagnosed with pN1 by SLNB when global ePLND is pN1), *specificity* (probability of being diagnosed with pN0 when global ePLND is pN0), *positive predictive value* (PPV), *negative predictive value* (NPV), *false negative* [(FN): SLN without metastasis while the ePLND reveals metastasis (3)], *false positive* [(FP): SLN containing metastasis outside the ePLND template while the ePLND does not reveal any metastasis (3)], *diagnostic yield* [(DY): patients where SLNB was successfully detected], and *non-diagnostic rate* [(NDR): patients where intraoperative gammaprobe did not detect any SLN] were calculated. Data on surgical complications were collected at 6 months after surgery according to the Clavien–Dindo classification (13).

## Results

The baseline clinical characteristics of the cohort are presented in Table 1. All patients were cT1–T3, ISUP/WHO grade group 1–5, and clinically localized (N0) in 95.3%, with a 17% mean probability of lymph node involvement according to the MSKCC nomogram.

**TABLE 1:** Clinical baseline characteristics.

	*n* (%)
Patients	64
Age, mean (IQR)	69.5 (62.3–72)
PSA, mean (IQR)	11.6 (6.7–17.8)
**Clinical stage (T)**	
T1c	20 (31.3)
T2	31 (48.4)
T3	13 (20.3)
**Clinical stage (*N*)**	
N0	61 (95.3)
N1	3 (4.7)
**Clinical stage (M)**	
M0	64 (100)
M1	0 (0)
**ISUP/WHO grade group**	
1	2 (3.1)
2	21 (32.8)
3	16 (25)
4	10 (15.6)
5	15 (23.4)
**Pattern**	
Acinar	45 (70.3)
Intraductal	19 (29.7)
MSKCC lymph node involvement probability, mean (IQR)	17% (10–19.5)

The surgical outcomes are listed in Table 2. The procedure was safe and no blood transfusion was required in any case. Overall, 23 (35.9%) patients experienced any type of complication according to the Clavien–Dindo classification, but only two of these (3.1%) were directly associated with the SLNB procedure. One of them was a symptomatic urinary tract infection with transient elevation of creatinine levels related to TRUS radiotracer injection. The other is a ureteral injury that requires surgical repair and ureteral stenting during para-aortic SLN dissection. In this case, the pathology report showed that this single SLNB was metastatic, whereas ePLND was negative (a false positive case). The surgical procedure was completed in all the cases.

**TABLE 2:** Surgical outcomes.

Surgical time, min, mean (IQR)	210 (182–228)
Transfusion rate, *n* (%)	0 (0%)
Urethral catheterization, days, mean (IQR)	9.7 (8–12)
Intraoperative complications	
Urethral injury (repaired and stenting), *n* (%)	1 (1.6)
Obturator vein injury (ligation), *n* (%)	1 (1.6)
Postoperative complications (Clavien-Dindo)	
Any kind (I–IV), *n* (%)	23 (35.9)
Grade I, *n* (%)	
Femoral parestesia (temporally), *n* (%)	5 (7.8)
Wound infection, *n* (%)	2 (3.1)
Transient elevation of serum creatinine, *n* (%)	1 (1.6)
Grade II, *n* (%)	
Symptomatic UTI, *n* (%)	4 (6.2)
Epidydimitis	1 (1.6)
Grade IIIa, *n* (%)	
Lymphedema	5 (7.8)
- Lymphedema + deep vein thrombosis	1 (1.6)
- Bladder neck stricture	1 (1.6)
- Ureteral stenting	1 (1.6)
Grade IIIb, *n* (%)	
Wound dehiscence	2 (3.13)
Grade IVb, *n* (%)	
Miastenia crisis	1 (1.6)
Pathological stage (T), *n* (%)	
T2	19 (29.7)
T3	45 (70.3)
Pathological stage (*N*), *n* (%)	
N0	46 (71.9)
N1	18 (28.1)
Prostatectomy margin status, *n* (%)	
Negative	41 (643.1)
Positive	234 (35.96.9)
Length positive margin (mm), *n* (%)	
<1	12 (18.85)
1–3	56 (7.89.2)
>3	6 (9.49.2)
SLN: sentinel lymph nodes	
UTI: urinary tract infection	

**FIGURE 3:**
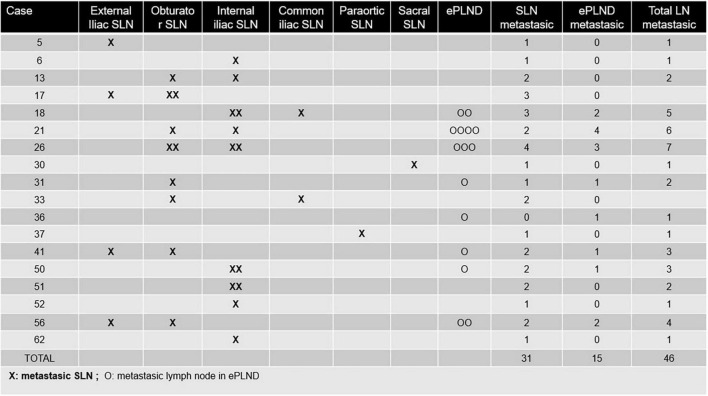
Regional distribution of LN metastasis. X, metatstasic SLN; O, metastasic lymph node in ePLND.

Final pathology revealed 18 (28.1%) patients with nodal metastasis. Mean number of lymph nodes ressected was 20.6 (IQR 15–24) with 3.3 SLN per patient (IQR 2–4.2) 26 SLN were located and dissected outside the ePLND template; 46/1,316 (3.5%) lymph nodes had nodal metastases, and 31/46 (67.4%) were SLNs (Figure 3). These findings were correlated at the patient level (Table 3), with 94.4% sensitivity, 100% specificity, 100% PPV, 97.8% NPV, 5.5% FN (1 patient with negative SN whilst cancer was found in other ePLND), and 4.3% FP (2 patients with positive SN outside the ePLND template while the ePLND template was negative) (see Supplementary Table 1). In four patients, no SLN was detected on lymphoscintigraphy, but thereafter, the gamma probe detected SLN in one of those three cases, leading to an NDR of 4.7% and a DY of 95.3%. Ten out of 18 pN + cases (56%) only had lymph nodal involvement in the SLNB.

**TABLE 3:** Sentinel lymph nodes and extended pelvic lymph node dissection findings.

Lesion level			
Lymph node dissection	*n* (%)	Mean	IQR
ePLND	1,102 (83.7)	17.2	13.5–22
SLN	214 (16.3)	3.3	2.2–4.2
Total	1,316 (100)	20.6	15–24.2
Total metastatic LN, *n* (%)			46 (100)
Metastatic SLN			31 (67.4)
Metastatic ePLND non-SLN			15 (32.6)
Number of SLN outside ePLND template			26/214 (12.1)
Patient level			
Sensitivity			94.4%
Specificity			100%
PPV			100%
NPV			97.8%
FN			5.5%
FP			4.3%
DY			95.3%
NDR			4.7%
Number of patients with metastasis to SLN only			10/18 (55.6%)

*LN, lymph nodes; SLN, sentinel lymph node; PPV, positive predictive value; NPV, negative predictive value; FP, False positive [SLN containing metastasis outside the ePLND template while the ePLND does not reveal any metastasis (3)]; FN, False negative [SLN without metastasis while the ePLND reveals metastasis (3)]; DY, Diagnostic yield (patients where SLNB was successfully detected); NDR, Non-diagnostic rate (patients where intraoperative gammaprobe did not detect any SLN).*

*Sensitivity, probability of being diagnosed of pN1 by SLNB when global ePLND is pN1.*

*Specificity, probability of being diagnosed of pN0 when global ePLND is pN0.*

The most frequent distribution of SLN was the obturator fossa (33.5%), internal iliac (22.5%), and external iliac (32.5%), while other sites represented a minor percentage (11.5%). The association between prostate-side injections and ipsilateral drainage was weak (Table 4). In only 26 (39%) cases, the SLN was coincident with the PCa tumor location (right-left-bilateral) and contralateral or bilateral drainage was observed more often.

**TABLE 4:** Topographic association between radiotracer side injection and SLN side findings.

Radiotracer prostate side injection	SLN location			
	
	Right (12)	Left (20)	Bilateral (29)	None (3)
Right (21)	6	2	12	1
Left (30)	4	14	11	1
Bilateral (13)	2	4	6	1

### Follow-Up

**FIGURE 4:**
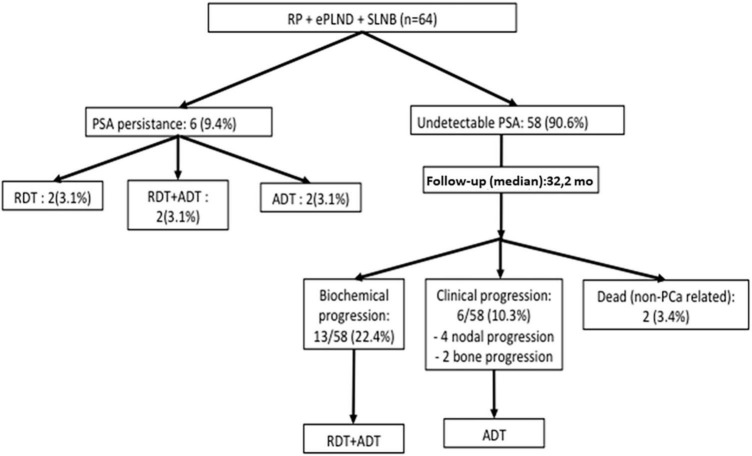
Follow-up flow-chart diagram. RP, radical prostatectomy; ePLND, extended pelvic lymphadenectomy; SLNB, sentinel lymph node biopsy; RDT, radiotherapy; ADT, androgen deprivation therapy.

The median follow-up period was 32.2 months (range 9.4–64.6). All cases with positive margins or node positivity were discussed by a multidisciplinary team for complementary treatments according to clinical considerations. PSA persistence was observed in six (9.4%) patients treated with radiotherapy (RDT) (*n* = 2), RDT + androgen deprivation therapy (ADT) (*n* = 2), or ADT (*n* = 2), depending on clinical considerations. Biochemical progression was observed in 13 (22.4%) cases, all of them treated with RDT and ADT. Six cases of clinical progression were observed: four lymph nodes and two metastatic bone progressions, treated with systemic therapies and targeted RDT in cases of oligometastatic disease. Two patients died of non-PCa-related causes (Figure 4).

Regarding pathological findings in lymphadenectomy, seven of the 18 cases of pN1 remained under observation: 4/18 (22.2%) with undetectable PSA levels and 3/18 (16.6%) with PSA < 0.1 ng/mL. The remaining 11 patients were deemed for salvage therapy, 10 with pelvic RDT + ADT and one case with ADT alone.

## Discussion

SLNB is an established procedure for several oncologic neoplasms, such as breast cancer, melanoma, or penile or vulvar cancer. However, the role of SLNB in PCa is still considered experimental, which may be explained, at least in part, by the heterogeneity and inconsistency of definitions, types of tracer, descriptions of interventions and detection methods (14). This issue has been assessed, and clear recommendations are stated in the literature (3, 8, 15) with current evidence showing the potential of SLNB for improving biochemical recurrence without increasing complications (6).

According to their principles, the radiotracer is injected into the whole gland based on the paradigm of the multifocality of PCa. However, within this multifocality, IL or primary tumor is known to drive the prognosis of PCa (10). Moreover, MRI has shown the capacity to detect IL and significant PCa foci (16). Taken together, we analyzed how an injection into the IL would eventually define the lymphatic drainage of the disease more precisely: with this approach, a 95.3% diagnostic yield was reached, with a sensitivity of 94.4% and specificity of 100%.

The prostatic injection technique of a radiotracer or indocyanine green-based fluorescence imaging in the published series is described as a bilateral injection to the peripheral prostate area, with 1–3 injections per side, without considering the location of the IL (17–25). Kjölhede et al. (24) performed a bilateral injection of the prostate by adding injections near the tumor, with an approximation to the selective injection with good results: 12% SLNB outside the ePLND template and a 4% false-positive rate. More recently, de Korne et al. demonstrated the benefit of intratumoural prostate tracer deposition, increasing the chance of visualizing nodal metastasis compared to extratemporal tracer deposition (26). Moreover, a prospective randomized phase II trial comparing intratumoural vs. intraprostatic injection demonstrated an increase in SLN-positive nodes (73%) in the intratumoural group compared to 37% in the intraprostatic group, although no metastasis-free survival differences were observed at a median follow-up of 47 months (27).

The diagnostic accuracy of SNLB for PCa was assessed in a systematic review by Wit et al. The overall test accuracy for all the studies included in the review revealed a 95.9% DY, 4.1% NDR, 95.2% sensitivity, 100% specificity, 100% PPV, 98% NPV, 0% FP, and 4.8% FN (3). Our results are at least equal to those of the bilateral non-selective approach to prostate injection, with better outcomes in terms of FP (4.3%). The false-positive rate may have an impact on patient outcomes, as it is referred to as the ability of the technique to detect the SLN outside the ePLND template and potentially excise nodal metastasis.

In contrast, the NDR was 4.7% (cases where no SLN was detected). All three were central transitional zone lesions. Special attention should be paid to cases of central lesions, and it could be preferable to perform a perilesional injection of tracer toward the peripheral area to ensure lymphatic drainage of the radiotracer.

In the present study, the anatomical pathways of lymphatic drainage in the PCa were found to be unpredictable (Table 4). These results are concordant with other evidence previously published in line with the aberrant lymphatic pathways of prostate tumors (28, 29). We suggest that there should be no right-left side considerations in cases where ePLND is performed, and surgical efforts should be the same on every pelvic side, irrespective of where the clinical information tells us that the tumor is located. Even in cases where the SLN is detected unilaterally, bilateral ePLND should be completed (9).

The rationale for this modification in the injection technique is based on the current evidence that IL in the PCa drive the natural history of the disease (10, 30). The identification and characterization of IL is mandatory and has improved with the widespread use of MRI and targeted prostate biopsies. Tan et al. demonstrated the capacity of MRI to detect IL, especially in cases of Gleason 7 or higher and 1 cm or larger PCa, with an 80% correlation with whole-mount prostate histopathology (31). There are treatment modalities, such as focal therapy based on the same principle of IL throughout MRI and targeted biopsy findings, with good long-term outcomes (32).

The oncological benefits of SLNB in ePLND remain controversial, but there is evidence indicating that SLNB in addition to ePLND improves biochemical recurrence-free survival compared with standard ePLND (3, 6, 7). Overall, with a mean follow-up of 33.1 months, 4/18 (22.2%) pN1 patients had undetectable PSA and 3/18 (16.6%) had PSA < 0.1 ng/mL. All these cases had a single metastatic nodal involvement, suggesting that patients with a limited burden of nodal metastasis have a significant chance of remaining free of biochemical recurrence at mid-term follow-up.

The main strengths of this study are the prospective design and our selected cohort of patients, with a specific and concise report of tumor characteristics that enables us to perform selective radiotracer injection. Moreover, our results show that targeted ^99^ *^m^*-Tc-nanocolloid injection into the IL is feasible, with excellent results, and that it follows the anatomical considerations of tumor spread. Based on these results, we present a modified option to assess SLNB in patients with PCa, switching from the paradigm of multifocality to the IL paradigm. These results are at least equivalent to whole gland injections.

The main limitation of the present study is the small number of cases. Increasing the cohort population size could enhance the consistency of the results. The study could also underestimate the intensity and number of SLNB; as the intensity of the ^99^ *^m^*Tc-nanocolloid decreases with time, our 2-day protocol is on the upper limit of reasonable time between injection and SLNB recommendations (3, 15). Furthermore, a control arm with a standard whole-gland injection was not included. Finally, preoperative assessment was performed with planar lymphoscintigraphy instead of SPECT/CT which has been demonstrated to be superior. Preoperative SPECT/CT after tracer injection can further improve the preoperative planning of LN dissection. Hybrid imaging could also have improved our results, as it permits better visualization of SLN and better detection in cases of deep abdominal SLN, and also reveals extra SLN near the prostate and outside the area of the ePLND (33).

## Conclusion

In conclusion, ultrasound-guided injection of ^99^ *^m^*Tc-nanocolloid into the prostate IL offers excellent results for staging in cases where SLND is considered. Further studies may confirm whether this selective approach will increase the diagnostic accuracy of SLNB in cases where the patient has an identifiable IL. This could provide a more precise selection of patients in need of ePLND without increasing the risk of relapse.

Patients with a limited burden of nodal metastasis have a significant chance of remaining free of biochemical recurrence at the mid-term follow-up.

## Data Availability Statement

The raw data supporting the conclusions of this article will be made available by the authors, without undue reservation.

## Ethics Statement

The studies involving human participants were reviewed and approved by the Institutional Ethical Committee: CEIm-Parc de Salut MAR code 8607. The patients/participants provided their written informed consent to participate in this study.

## Author Contributions

LF, JA, AM-F, and SV-S contributed to the conception, design of the study, and performed the statistical analysis. LF organized the database and wrote the first draft of the manuscript. JA, AM-F, SV-S, and NJ wrote sections of the manuscript. All authors contributed to manuscript revision, read, and approved the submitted version.

## Conflict of Interest

The authors declare that the research was conducted in the absence of any commercial or financial relationships that could be construed as a potential conflict of interest.

## Publisher’s Note

All claims expressed in this article are solely those of the authors and do not necessarily represent those of their affiliated organizations, or those of the publisher, the editors and the reviewers. Any product that may be evaluated in this article, or claim that may be made by its manufacturer, is not guaranteed or endorsed by the publisher.
