# Supplementary material for: Sentinel Lymph Node Biopsy in Prostate Cancer Patients: Results From an Injection Technique Targeting the Index Lesion in the Prostate Gland
Source: Front Med (Lausanne). 2022 Sep 2;9:931867. doi: 10.3389/fmed.2022.931867 (PMC9478858; doi:10.3389/fmed.2022.931867)
Supplement: Supplementary file 1 [file Table_1.DOCX]

Supplementary Table 1

Diagnostic accuracy indices calculation:

| pN0= 46/64  pN1=18/64 | | ePLND | |
| --- | --- | --- | --- |
|  |  | + | - |
| SLNB | + | 17 (TP) | 0 (FP) |
|  | - | 1 (FN) | 46 (TN) |

Sensitivity = TP/(FN+TP) = 17/(17+1)= 94.4%

Specificity = TN/(TN+FP) = 46/(46+0) = 100%

NPV= TN/(FN+TN) = 46/(46+1) = 97.8%

VPP=TP/(TP+FP) = 17/(17+0) = 100%

False negative (FN) = 1 patient with negative SN whilst cancer was found in other LN(ePLND) = 1/18 = 5.5%

False positive (FP) = 2 patients with positive SN outside the ePLND template while the ePLND template was negative= 2/46 = 4.3%

Diagnostic yield (DY) = patients where SLNB was sucessfully detected = 61/64 = 95.3%

Nondiagnostic rate (NDR)= patients where no SLN was detected = 3/64 = 4.7%
